# A case of refractory tumor bleeding from an ampullary adenocarcinoma: Compression hemostasis with a self‐expandable metallic stent

**DOI:** 10.1002/deo2.23

**Published:** 2021-08-22

**Authors:** Kazuma Daiku, Kenji Ikezawa, Shingo Maeda, Yutaro Abe, Yugo Kai, Ryoji Takada, Takuo Yamai, Nobuyasu Fukutake, Tasuku Nakabori, Hiroyuki Uehara, Kazuyoshi Ohkawa

**Affiliations:** ^1^ Department of Hepatobiliary and Pancreatic Oncology Osaka International Cancer Institute Osaka Japan

**Keywords:** ampullary tumor, anchoring stent, duodenal papillary carcinoma, refractory hemorrhage, stent migration

## Abstract

Although patients with ampullary cancers frequently experience obstructive jaundice and tumor bleeding, there have been few reports on efficient management of refractory hemorrhage after conservative treatment. In this report, we describe a case of refractory bleeding from a 15‐mm ampullary adenocarcinoma. A Japanese woman in her 60s was urgently hospitalized for cholangitis, pancreatitis, and sepsis treatment. Investigation with a side‐viewing duodenoscope revealed an ulcerated ampullary adenocarcinoma. After the patient underwent anticoagulation therapy for pulmonary thromboembolism, the tumor bleeding gradually increased, resulting in severe anemia. Because the anemia did not improve with fasting or discontinuation of the anticoagulation therapy, the patient underwent repeated red blood cell transfusions. As no hemobilia was observed in the bile juice aspirated during endoscopic retrograde cholangiography, we supposed that the bleeding originated from the ulcerative cancer surface. We did not perform thermal therapy because we considered that it would worsen the bleeding. Abdominal angiography showed no pseudoaneurysms or extravasation. Ultimately, we performed transpapillary placement of a fully covered self‐expandable metallic stent (SEMS) with an anchoring double pigtail plastic stent that resulted in successful hemostasis. In this case, the mechanism of hemostasis was not presumably explained by direct compression of the bleeding point but by indirect compression. When tumor volume is small, the radial force of the SEMS may cause compression of the tumor volume, leading to shrinkage of the bleeding blood vessels. In conclusion, covered SEMS placement could be an efficient treatment for refractory ampullary cancer bleeding, even from an ulcerated cancer surface.

## INTRODUCTION

Ampullary cancers comprise approximately 16%–28% of periampullary cancers.^1^ Advanced ampullary cancers frequently cause obstructive jaundice and bleeding.^1^ To manage the obstructive jaundice, transpapillary biliary decompression is performed with plastic and self‐expandable metallic stents (SEMSs).^2^ Contrastingly, a standard approach to hemostasis for ampullary cancers is yet to be determined. There are limited data on the most efficient treatments for refractory hemorrhage following conservative treatment of patients with ampullary cancer. Radical surgical resection (pancreatoduodenectomy) provides definite hemostasis^3^; however, it is invasive and cannot be applied to high‐risk patients such as older patients, those with severe comorbidities, and obese patients. Here, we present a case of refractory tumor bleeding from an ampullary cancer treated by transpapillary SEMS placement and describe its efficiency as a method of hemostasis through indirect compression of a bleeding point by the radial force of the SEMS.

## CASE REPORT

**FIGURE 1:**
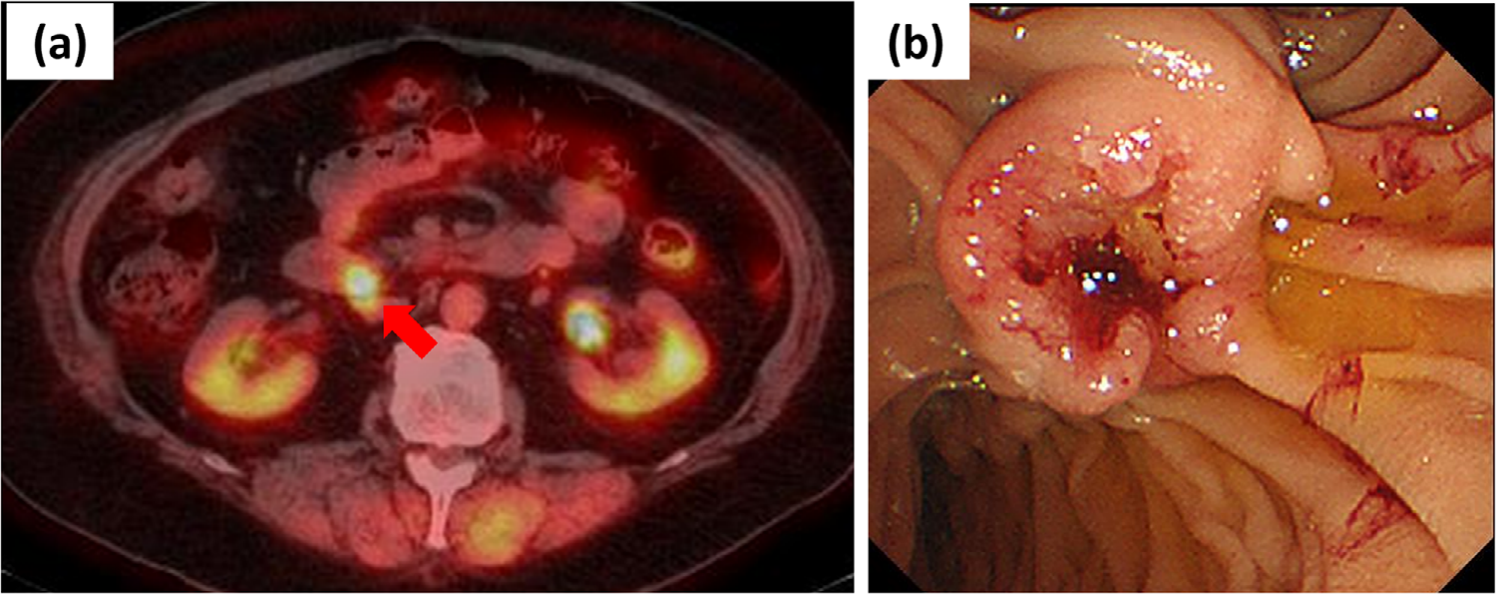
(a) Positron emission tomography/computed tomography scan, in which ^18^F‐fluorodeoxyglucose uptake was seen around the papilla of Vater (red arrow). (b) Examination with the side‐viewing duodenoscope, revealing a 15‐mm, ulcerated tumor on the papilla of Vater

A Japanese woman in her 60s with type 2 diabetes and obesity was followed up at our facility after undergoing chemotherapy for uterine sarcoma. She underwent a positron emission tomography/computed tomography (CT) scan to further investigate the reason for her elevated CA19‐9 level (136 U/ml), during which ^18^F‐fluorodeoxyglucose uptake was seen around the papilla of Vater (Figure 1a). Before this investigation, she was urgently hospitalized for examination and treatment of fever and impaired awareness. There was a marked increase in her total bilirubin, hepatobiliary and pancreatic enzymes, D‐dimer, and lactate (Table 1).

**TABLE 1:** Laboratory data on admission

Blood cell count		Biochemical data	
WBC (/μl)	5660	Total Protein (g/dl)	6.8
Hemoglobin (g/dl)	14.1	Albumin (g/dl)	4.0
Platelets (× 10^4^/μl)	18.2	T‐Bilirubin (mg/dl)	1.7
Neutrocytes (%)	93.7	Aspartate transaminase (U/L)	592
Arterial blood gas		Alanine transaminase (U/L)	504
pH	7.448	Alkaline phosphatase (U/L)	374
pO₂ (mm Hg)	103	γ‐glutamyltransferase (U/L)	412
pCO₂ (mm Hg)	27.4	Amylase (U/L)	2210
HCO3‐ (mEq/L)	18.7	Lactate dehydrogenase (U/L)	750
Base excess (mEq/L)	−3.6	Creatinine kinase (U/L)	34
Lactate (mmol/L)	4.1	Blood urea nitrogen (mg/dl)	17
Coagulation test		Creatinine (mg/dl)	0.69
aPTT (sec)	22.2	Sodium (mmol/L)	139
PT (%)	95	Potassium (mmol/L)	3.9
PT‐INR	1.02	Chloride (mmol/L)	102
D‐Dimer (μg/ml)	14.2	Calcium (mg/dl)	9.3
		C‐reactive protein (mg/dl)	1.82

Abbreviations: aPTT, activated partial thromboplastin time; INR, international normalized ratio; PT, prothrombin time; T‐Bilirubin, total bilirubin; WBC, white blood cell.

**FIGURE 2:**
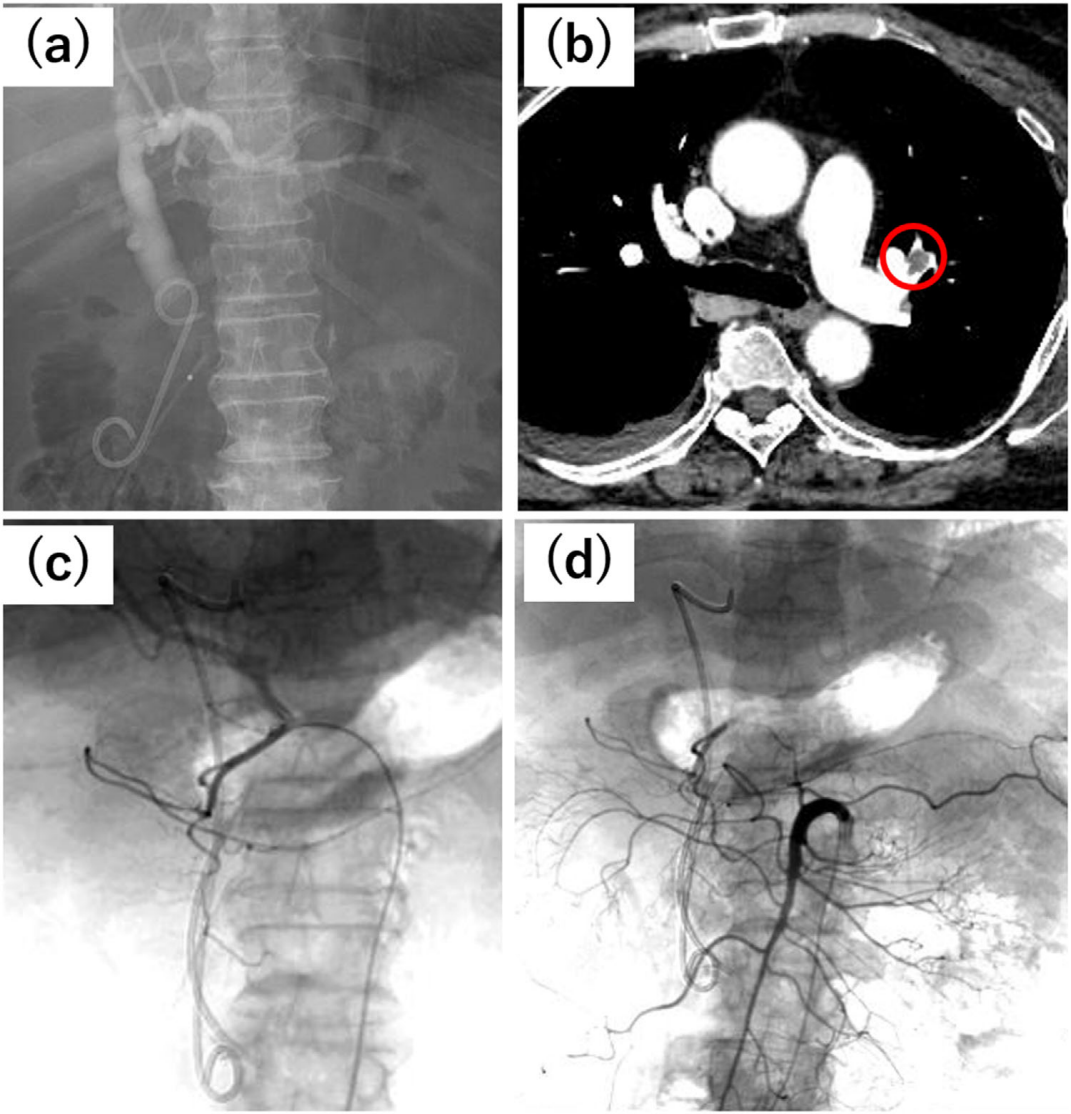
(a) We placed 7‐French (Fr) double pigtail and 5‐Fr straight stents for the biliary and pancreatic duct obstruction, respectively. (b) Contrast‐enhanced computed tomography revealed a thrombus in the left pulmonary artery (red circle). (c) Angiography of the gastroduodenal artery and (d) superior mesenteric artery, showing no pseudoaneurysm or extravasation

After undergoing conservative treatment in the intensive care unit for the diagnoses of severe cholangitis, pancreatitis, and sepsis, she underwent side‐viewing duodenoscopy, which found a 15‐mm, ulcerated tumor on the papilla of Vater (Figure 1b). Adenocarcinoma was confirmed by forceps biopsy. A 7‐French (Fr) double pigtail stent (Medi‐Globe, Achenmühle, Germany) was placed to treat the obstructive jaundice, while a 5‐Fr straight stent (Advanix Pancreatic Stent; Boston Scientific, Marlborough, United States) was placed to manage the pancreatic duct obstruction (Figure 2a).

**FIGURE 3:**
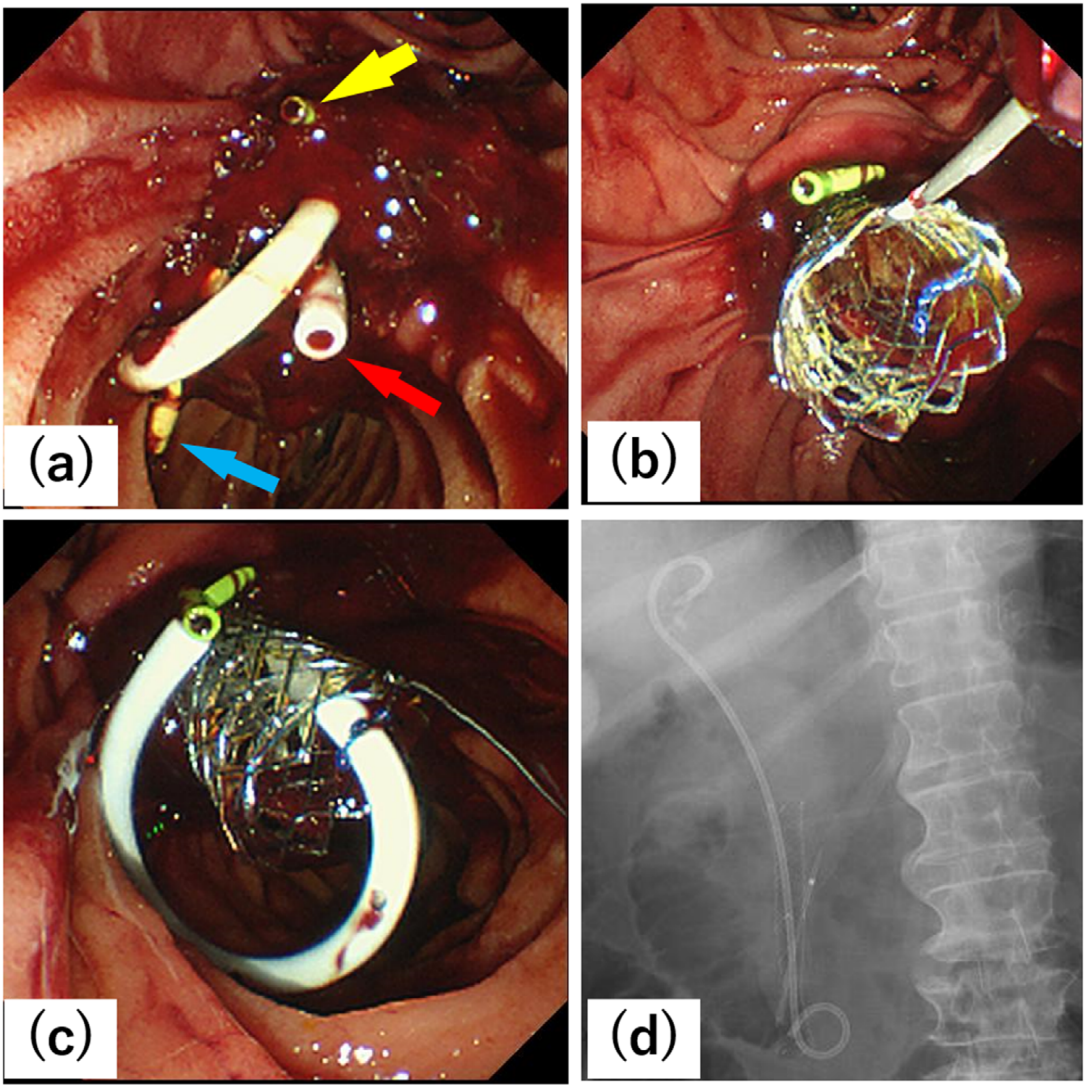
(a) Blood clot attached to the ampullary cancer on side‐viewing endoscopy on the 48th day of hospitalization. The double pigtail and straight plastic stents (red and blue arrows, respectively) for biliary obstruction were observed. The pancreatic stent (yellow arrow) was also observed. (b) Transpapillary placement of a 10‐mm fully covered self‐expandable metallic stent (SEMS). (c) A 7‐French double pigtail plastic stent was placed as an anchoring stent. (d) Fluoroscopic image of the fully covered SEMS placement with anchoring plastic and pancreatic stents

Contrast‐enhanced CT on the 20th day of hospitalization found a pulmonary thromboembolism (PTE) (Figure 2b), which was treated with continuous intravenous heparin followed by a direct oral anticoagulant (DOAC) from the 37th day of hospitalization. On the 40th day, an endoscopic nasobiliary drainage (ENBD) tube was placed due to biliary stent occlusion. Four days later, we performed transpapillary placement of multiple plastic stents (a 7‐Fr double pigtail plastic stent and a 7‐Fr straight plastic stent) because the ENBD tube had migrated distally. From the 45th day of hospitalization, she suffered from anemia due to the tumor bleeding, which did not improve with fasting or DOAC discontinuation. She required repeated red blood cell transfusions (minimum hemoglobin level, 5.1 g/dL). The side‐viewing duodenoscopy on the 47th day of hospitalization found a blood clot attached to the ampullary cancer. Thermal therapy, including argon plasma coagulation (APC), was not performed because it is associated with a risk of worsened bleeding. On the 48th day, due to continued hemorrhage, she underwent angiography for arterial embolization, which found no pseudoaneurysms or extravasation (Figures 2c–2d). The surgeons did not recommend performing pancreatoduodenectomy for hemostasis due to her poor performance status and severe obesity. On the same day (the 48th day of hospitalization), side‐viewing endoscopy was repeated, which revealed a blood clot attached to the ampullary cancer (Figure 3a). We supposed the bleeding was from: 1) the common bile duct in the tumor and 2) the ulcerated tumor surface. Endoscopic retrograde cholangiography found no filling defect in the bile duct. In addition to noting the absence of obvious hemobilia in aspirated bile juice, bleeding from the ulcerated tumor surface was considered the primary cause of the hemorrhage. We expected that the radial force of an SEMS would compress the tumor volume because the tumor was small; thus, transpapillary placement of a 10‐mm fully covered SEMS (Bonastent, Standard Sci Tech, Seoul, Korea) was performed (Figure 3b, Video S1). During previous hemostatic procedures, blood oozing was observed from the tumor surface, but it ceased after SEMS placement. Additionally, a 7‐Fr double pigtail plastic stent (Medi‐Globe, Achenmühle, Germany) was placed as an anchoring stent to decrease the risk of SEMS migration (Figures 3c–3d, Video S1). No additional bleeding was observed after these endoscopic procedures (Figure S1). After SEMS placement, no further red blood cell transfusion was required. The patient was discharged on the 67th day of hospitalization. The pancreatic stent occlusion occurred 2 months after discharge. Until then, the biliary stent migration and recurrent bleeding from the ampullary cancer had not been observed.

## DISCUSSION

In this report, refractory bleeding from an ulcerated duodenal papillary adenocarcinoma was treated with transpapillary SEMS placement. Of all ampullary cancer patients, 33% experience gastrointestinal bleeding that is chronic and frequently occult.^1^ Contrastingly, there have been few reports on the approach to hemostasis in ampullary cancers, with bleeding that was not resolved through conservative treatments. Although antiplatelets and anticoagulants increase the risk of gastrointestinal bleeding,^4^ in our case, the anemia worsened rapidly even after discontinuation of the DOAC used to treat the PTE. Based on the finding of no hemobilia in the aspirated bile juice and ENBD tube, we suspected that the bleeding had originated from the ulcerated ampullary cancer surface. APC could potentially be used to treat gastrointestinal tumor bleeding; however, its efficacy in managing hemorrhage from ampullary cancers remains unclear. Moreover, its use may result in worsened bleeding by increasing the tumor tissue fragility. Emergent surgical resection (pancreatoduodenectomy) was also not performed due to the patient's poor general condition. Therefore, we performed transpapillary placement of a fully covered SEMS, expecting that it would compress the tumor volume. Several reports have shown that covered SEMS placement is useful for hemobilia associated with tumors (liver metastasis from rectal cancer)^5^ as well as bleeding after endoscopic biliary sphincterotomy.^6^ The mechanism of hemostasis is direct compression of the hemorrhage origin by the radial force of the large diameter covered by the SEMS. In contrast, there have been no reports on SEMS placement's efficacy for hemorrhage from ampullary cancers. The mechanism of hemostasis cannot be fully explained by direct compression of the bleeding point, because there was no clear evidence to suggest hemobilia. In addition to the finding that blood oozing from tumor surface ceased after SEMS placement, the mechanism can be explained by indirect compression. In small tumors, it is presumed that the radial force of the SEMS could cause compression of tumor volume, leading to shrinkage of the bleeding blood vessels that are exposed to the ulcerated surface.

Although a meta‐analysis found that uncovered SEMSs have a lower risk of migration than covered SEMS,^7^ we chose to place a covered SEMS to prepare for future exchange of a pancreatic plastic stent. Fully covered SEMS in patients with distal malignant biliary obstruction (MBO) have a high risk of migration.^8^ Furthermore, in this case, stent migration may have caused the recurrent bleeding. Considering these risks, we also placed a double pigtail plastic stent as an anchoring stent for anti‐migration. Previous studies have described the efficacy of anchoring stents in patients with benign biliary strictures^9^ and in those with MBO.^10^ Fully covered SEMS placement in patients with ampullary cancer may increase the migration risk due to a short biliary stricture. Fortunately, our patient had an uneventful course with no stent migration. Adding an anchoring plastic stent after fully covered SEMS placement could be useful in decreasing the migration risk in patients with ampullary cancer.

In conclusion, covered SEMS placement could be a useful treatment option for refractory ampullary cancer hemorrhage, even from an ulcerative surface. This report highlights a potentially novel role for covered SEMS in indirect compression hemostasis.

## CONFLICT OF INTEREST

The authors declare no conflict of interest for this article.

## ETHICS STATEMENT

All procedures performed in studies involving human participants were in accordance with the ethical standards of the institutional and/or national research committee and with the 1964 Helsinki declaration and its later amendments or comparable ethical standards. Written informed consent was obtained from the patient for publication of this case report and any accompanying images.

## FUNDING INFORMATION

None.

## Supporting information


**Supplementary Figure S1**: Successful hemostasis was confirmed on observation with the side‐viewing duodenoscope on the 53^rd^ day of hospitalization.

**Video S1**: Refractory hemorrhage from an ampullary cancer was successfully treated with transpapillary placement of a fully covered self‐expandable metallic stent (SEMS) and an anchoring double pigtail plastic stent.
