# Supplementary figures and images for: A case of refractory tumor bleeding from an ampullary adenocarcinoma: Compression hemostasis with a self‐expandable metallic stent
Source: DEN Open. 2021 Aug 22;2(1):e23. doi: 10.1002/deo2.23 (PMC8828171; doi:10.1002/deo2.23)

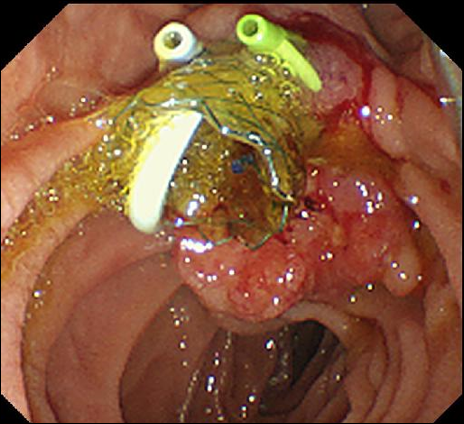

Supplement: Supplementary file 1 — Supplementary Figure S1: Successful hemostasis was confirmed on observation with the side‐viewing duodenoscope on the 53rd day of hospitalization. [file DEO2-2-e23-s002.tif]
